# Notch Signaling Pathway Regulates Ozone-Induced Lung Circadian Rhythm Disruption

**DOI:** 10.3390/toxics13090733

**Published:** 2025-08-30

**Authors:** Xinyu Zhang, Xiaotong Jian, Xinyi Miao, Yangyang Jia

**Affiliations:** 1 Henan Clinical Research Center of Childhood Diseases, Henan Children’s Hospital, Zhengzhou Children’s Hospital, Children’s Hospital Affiliated to Zhengzhou University, Zhengzhou 450018, China; 2 School of Public Health, Zhengzhou University, Zhengzhou 450001, China

**Keywords:** *Notch3*, *Notch4*, circadian rhythm, ozone, *Bmal1*, *Per*, air pollution

## Abstract

Background: Ozone (O_3_) pollution disrupts pulmonary circadian rhythms, yet the molecular mechanisms remain elusive. The Notch signaling pathway, critical for lung homeostasis, may crosstalk with the circadian clock system. Objective: This study elucidates the role of the Notch signaling pathway in O_3_-induced lung circadian rhythm disruption. Methods: C57BL/6J mice were acutely exposed to O_3_ (1.0 ppm, 3 h). Lung tissues were collected 24 h post exposure. Transcriptome sequencing coupled with GSEA identified dysregulated pathways; IHC and RT-qPCR validated core genes; GEO dataset (GSE58244) reanalysis assessed *Notch3/4* knockout effects. Results: O_3_ activated Notch signaling (NES = 1.85, FDR = 0.034) and disrupted the circadian pathway (NES = 1.84, FDR = 0.029), downregulating *Bmal1* while upregulating *Per2/3* and *Notch3/4* (*p* < 0.05). Strong correlations (r > 0.8) existed between core genes of both pathways. *Notch3/4* knockout exacerbated circadian disruption in a time-dependent manner upon O_3_ exposure. Conclusion: O_3_ induces lung circadian disruption via *Notch3/4* activation, which provides novel mechanistic insights into pollutant-induced lung injury.

## 1. Introduction

Tropospheric ozone (O_3_) is a secondary air pollutant formed through photochemical reactions between nitrogen oxides (NO_x_) and volatile organic compounds (VOCs). This process is driven by factors such as climate [1,2] and human activities [3,4], with well-documented negative impacts on human health. The data from the Global Burden of Disease (GBD) study highlight the severity of its health toll: in 2019, O_3_ pollution was linked to 365,222 deaths, accounting for 0.65% of global disability-adjusted life years (DALYs) [5]. Current estimates indicate that by 2025, 66.2% of the global population will be exposed to excessive O_3_ in the short term, while 94.2% will be exposed to O_3_ over an extended period [6].The low molecular weight of O_3_ (only 4.8 g/mol) and its high water solubility enable it to penetrate deep into the airways, where it can come into direct contact with the lining fluid or cell membranes in the bronchiolar and alveolar wall regions, causing inflammatory injury to the respiratory tract [7].Clinical evidence further supports these detrimental effects: acute exposure to O_3_ at levels close to ambient concentrations (120 ppb) has been shown to reduce lung function and induce airway damage and inflammation in older adults [8]. Moreover, Long-term exposure to O_3_ has also been clearly associated with the development of asthma and acute exacerbations [9]. Therefore, a comprehensive understanding of these health risks is critical for addressing the growing challenges posed by O_3_ pollution.

The lung is not a static organ passively enduring damage, but possesses an endogenous circadian rhythm that is relatively independent of the central clock [10]. This rhythm is essentially a 24 h periodic molecular oscillation driven by core circadian rhythm genes (such as *Bmal1*, *Clock*, *Per*, and *Cry*) through a trilateral transcriptional-translational feedback loop system [11], which in turn regulates diurnal fluctuations in physiological processes including immune responses [12,13], oxidative stress [14,15], and cellular repair [10,16]. Disruption of the circadian rhythm in lung tissue is closely associated with respiratory diseases. Clinical studies have clearly demonstrated that patients with asthma and Chronic obstructive pulmonary disease (COPD) exhibit abnormal expression of rhythm genes, accompanied by molecular characteristics of exacerbated nighttime symptoms [17,18,19]. Animal experiments further confirm that circadian rhythm disruption or deletion of core circadian rhythm genes accelerates the progression of pulmonary diseases such as lung cancer [20], COPD [21], and asthma [22]. In this context, the potential harm of air pollutants such as O_3_ may not be limited to direct oxidative damage, but also includes interference with the circadian rhythm of lung tissue. Currently, there is evidence that exposure to air pollutants (e.g., O_3_ [23], PM_2.5_ [24,25], and benzo-[a]-pyrene [26]) can induce circadian rhythm disruption. However, the mechanism by which O_3_ interferes with the pulmonary circadian rhythm remains at the level of phenomenological description, and the specific molecular pathways are unknown.

The key to elucidating the molecular mechanism by which O_3_ disrupts circadian rhythms lies in identifying the common core signaling pathway linking O_3_ exposure and rhythm disturbances. As a critical regulatory pathway for lung tissue development and homeostasis, Notch signaling may play a pivotal role in this process [27]. The Notch signaling pathway is a widespread and conserved signaling system, whose receptor-ligand interactions are extensively involved in regulating the progression of diseases characterized by circadian rhythm disorders, such as asthma [28,29], COPD [30], and lung cancer [31]. More importantly, crosstalk between the Notch signaling pathway and the circadian rhythm system has been confirmed in studies across multiple fields, including ischemic stroke [32], bone regeneration [33], and intestinal stem cells [34]. Additionally, co-enrichment of both pathways has been observed in Drosophila exposed to pollutants [35]. This suggests that air pollutants may affect the Organism’s rhythmic balance by disrupting the crosstalk between Notch signaling and circadian rhythms. Furthermore, studies have reported that O_3_ exposure alters the expression of Notch receptors, and deletion of the *Notch3* or *Notch4* gene significantly exacerbates O_3_-induced pulmonary inflammation [36]. Based on this, the present study aims to investigate the regulatory role of the Notch signaling pathway (particularly *Notch3/4*) in core circadian rhythm genes during O_3_ exposure-induced circadian rhythm disruption in lung tissue.

Based on the above background and scientific questions, this study established a mouse model of acute O_3_ exposure (1.0 ppm, 3 h/d) [37]. Combining transcriptome sequencing to analyze changes in transcriptional characteristics, we verified the expression changes in circadian rhythm genes and key molecules in the Notch signaling pathway in lung tissues using Reverse Transcription Quantitative PCR (RT-qPCR) and immunohistochemistry (IHC), and clarified the correlation between the two. On this basis, we utilized a Gene Expression Omnibus (GEO) dataset (lung tissue data from O_3_-exposed *Notch3/4* knockout mice) [36] to verify the impact of *Notch3/4* deficiency on the expression of circadian rhythm genes, thereby further exploring the molecular mechanism by which O_3_ induces circadian rhythm disruption through the Notch signaling pathway. This study aims to clarify the critical role of the Notch signaling pathway in O_3_-induced circadian rhythm disruption in lung tissues, provide new theoretical basis for understanding the time-dependent mechanism of air pollutant-induced respiratory system damage, and simultaneously offer potential targets for the development of protective strategies targeting rhythm regulation.

## 2. Materials and Methods

### 2.1. Animals and Exposure

Seven- to eight-week-old male C57BL/6J mice (SPF grade, *n* = 12, 23–27 g) were randomized to two groups (*n* = 6/group). All animals were housed under controlled conditions (24 ± 1 °C, 55 ± 5% humidity) with 12 h/12 h light-dark cycles (lights on at 8:00), provided ad libitum access to sterile rodent chow and autoclaved water. The O_3_ group received a single 3 h ozone exposure (1.0 ppm) using an animal exposure system (TOW Intelligent Technology Co., Ltd. Shanghai, China) from 14:00 to 17:00. This dosage has been confirmed to induce pulmonary inflammatory injury in animals [37]. For the control group, mice were subjected to the same experimental conditions but exposed to filtered air (FA) instead of ozone. At 24 h post exposure, two group mice were anesthetized with sodium pentobarbital (100 mg/kg, intraperitoneal injection) and lungs were harvested. All animal experiments in our study were performed in accordance with institutional guidelines and were approved by the Experimental Animal Ethics Committee of Zhengzhou University (Approval Protocol No. ZZUIRB2023-144).

### 2.2. RNA Extraction and Quality Assessment

Total RNA was isolated from pulverized lung tissue using TRIzol™ Reagent (Servicebio, Wuhan, China) with chloroform purification. RNA integrity was verified through spectrophotometry (Nano Drop 2000: A260/A280 = 1.8–2.1, A260/A230 ≥ 2.0) and bioanalyzer assessment (Agilent 2100, RNA Integrity Number ≥ 7). Qualified samples proceeded to poly(A) selection using Oligo(dT) beads prior to library construction. Transcriptome Library Construction and Sequencing. Libraries were prepared per NEBNext^®^ Ultra™ II protocol (E7770S, New England Biolabs is headquartered in Ipswich, MA, USA) involving mRNA fragmentation (200 bp), cDNA synthesis with M-MuLV reverse transcriptase, end-repair/A-tailing, and Illumina adapter ligation. Size-selected libraries (AMPure XP beads) were quantified via Qubit 3.0 and validated by Agilent 2100 prior to 150 bp paired-end sequencing on Illumina Nova Seq 6000 (Gene Denovo Biotechnology, Guangzhou, China) at 40 million reads/sample.

### 2.3. Differentially Expressed Genes (DEGs) Analysis

Differential gene and functional enrichment analyses were performed to identify changes in the circadian rhythm and Notch signaling pathway following ozone exposure. Raw reads were trimmed (Trimmomatic v0.39) and filtered (Q-score > 20, N-content < 10%). Clean reads were aligned to GRCm39 using STAR v2.7.10b (splicing-aware mode). Gene expression was quantified as FPKM (Cufflinks v2.2.1) with differential expression analysis via DESeq2 v1.38.3 [|log_2_ FC| ≥ 1.5, False Discovery Rate (FDR) < 0.05]. Functional enrichment was performed on Omicsmart system (v3.0).

### 2.4. Protein–Protein Interaction (PPI) Network Construction

Genes from the Circadian rhythm and Notch signaling pathway were imported into the STRING database [38] to identify hub genes functioning critically within these pathways (minimum required interaction score: highest confidence > 0.9). Visualization was performed using Cytoscape (v3.10.3), and Pearson correlation analysis was applied to assess interactions among these key genes.

### 2.5. Immunohistochemistry

The left lungs of mice (3 per group) were fixed in 4% paraformaldehyde for 24 h, followed by paraffin embedding. IHC was performed according to the method described by Mengyuan Li et al. [39]. The antibodies utilized in this study were BMAL1 (Proteintech Cat No. 14268-1-AP, Proteintech Group, Inc., Rosemont, IL, USA), PER2 (Thermo cat PA5-100107, Shanghai, China), PER3 (Proteintech cat 12550-1-AP), Basic Helix-Loop-Helix Family Member E40 (BHLHE40) (Thermo cat PA1-16546), NOTCH1 (Wanleibio cat WL03097, Wanleibio Co., Ltd., Shanghai, China), NOTCH2 (Wanleibio cat WL02409), NOTCH3 (Servicebio cat GB112035-100, Wuhan, China) and NOTCH4 (Wanleibio cat WL00379) primary antibodies. Positive staining areas were quantified in ≥3 fields/sample using ImageJ v1.53 (Consistent magnification for each field of view), with area fraction = (positive pixels/total tissue pixels) × 100%.

### 2.6. Reverse Transcription Quantitative PCR Validation

Total RNA was re-extracted from mice’s right lungs (3 per group) using the same method. RT-qPCR was performed for in vivo validation of key pathway genes. Total RNA was reverse-transcribed (PrimeScript RT Kit) and amplified (SYBR Green) with primersfor *Bmal1*, *Bhlhe40*, *Per2*, *Per3* and *Notch1/2/3/4* Relative expression calculated via 2^−ΔΔCt^ method. Primers were synthesized by Sangon Biotech (Shanghai, China). The primer sequences are provided in Table 1.

### 2.7. GEO Data Acquisition and Source

To verify the regulatory effect of the Notch signaling pathway on circadian rhythms, we identified a relevant dataset from the GEO database. (accession number: GSE58244) [36]. This dataset was conducted by Dr. Steven Kleeberger’s team at the National Institute of Environmental Health Sciences (NIEHS), encompasses gene expression profiles derived from lung tissues of wild-type (WT), *Notch3* knockout (*Notch3*−/−), and *Notch4* knockout (*Notch4*−/−) mice (aged 7–13 weeks) exposed to either filtered air (control) or 0.3 ppm O_3_ for durations of 6, 24, or 48 h. Three biological replicates per genotype per exposure group were analyzed using the Affymetrix GeneChip Mouse Genome 430 2.0 Array. For the current analysis focusing on O_3_-induced Notch signaling alterations, we specifically extracted data from O_3_-exposed groups and their corresponding air-exposed controls across all genotypes and time points.

### 2.8. Gene Set Enrichment Analysis

Genome-wide expression profiles from the GSE58244 dataset were subjected to Gene Set Enrichment Analysis (GSEA, v4.4.0) to identify O_3_-perturbed (KEGG) pathways across genotypes [40]. A custom KEGG gene set collection was curated from the KEGG Database, with targeted interrogation of the circadian rhythm pathway (KEGG ID: ko04710). Analyses were stratified by genotype into the following groups: wild-type (WT) control mice, *Notch3*-knockout mice, and *Notch4*-knockout mice. To validate prior findings, expression levels of core circadian rhythm genes were extracted from the normalized expression matrix. Box plots were generated for visualized comparisons.

### 2.9. Statistical Analysis

Statistical analyzes were performed using GraphPad Prism 8. Data are presented as mean ± SEM. Unpaired Student’s *t*-test (two-tailed, α = 0.05) was used for comparisons between two groups. Differential genes (|log_2_FC| ≥ 1.5, *p* < 0.05) were identified via DESeq2 v1.38.3. Pearson correlation, GSEA (Normalized Enrichment Score (NES) > |1.5|, FDR < 0.05; v4.4.0), and CytoNCA v2.1.6 (for PPI network) were applied. Heatmaps were generated using R v4.2.0. Graphs were plotted with GraphPad Prism 8 *p* < 0.05 was significant.

## 3. Result

### 3.1. Transcriptomic Profiling Revealed Profound O_3_-Induced Alterations in Murine Lungs

**Figure 1:**
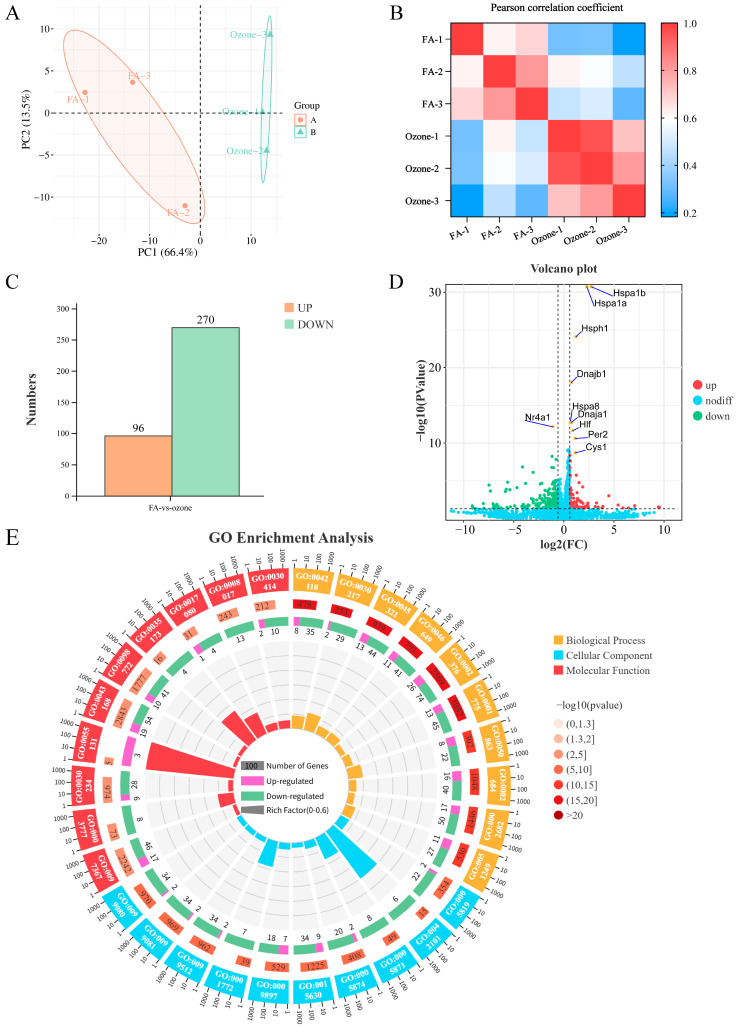
Transcriptomic profiling of murine pulmonary responses to O_3._ (**A**) Principal Component Analysis (PCA) of RNA-seq data. Axes indicate variance explained. FA: filtered air (orange; *n* = 3). Ozone: 1.0 ppm exposed (green; *n* = 3). (**B**) Pairwise Pearson correlation matrix. Sample IDs: FA1–3, Ozone1–3. (**C**) Bar plot of differentially expressed genes (DEGs). Thresholds: FDR < 0.05, |log_2_FC| > 1.5 (**D**) Volcano plot of DEGs. Red: upregulated; green: downregulated; gray: non-significant. Top 10 significant DEGs labeled. (**E**) Circular plot of Gene Ontology (GO) enrichment. Ontologies: Biological Process (BP, yellow), Cellular Component (CC, blue), Molecular Function (MF, red). Rings display: (1) GO term IDs; (2) background gene counts; (3) DEG counts (upregulated: purple, downregulated: green); (4) gene ratio.

PCA demonstrated global segregation of samples by exposure status (Figure 1A), with filtered air (FA) controls (orange) and O_3_-exposed specimens (green) forming discrete clusters along PC1 (66.4% variance) and PC2 (13.5% variance). This divergence was corroborated by Pearson correlation analysis (Figure 1B), showing high intra-group homogeneity versus significant inter-group divergence. Differential expression analysis identified 366 significantly altered genes (Figure 1C) under stringent thresholds (|log_2_FC| > 1.5, *p* < 0.05), comprising 270 downregulated (green) and 96 upregulated (orange) transcripts. The volcano plot (Figure 1D) highlighted top-ranked DEGs driving this response, including heat shock regulators (*Hspa1b*, *Hspa1a*, *Hsph1*, *Dnajb1*, *Hspa8*, *Dnaja1*), circadian-related factors (*Nr4a1*, *Hlf*, *Per2*), and Cilia-associated protein coding gene (*Cys1*) showing significance regulation. Functional enrichment analysis showed that O_3_ exposure mainly disrupted immune-related pathways (Figure 1E). As indicated by Gene Ontology (GO) terms, the top three significantly enriched entries in Biological Process (BP), Molecular Function (MF) and Cellular Component (CC) categories were: for BP: [GO:0030217_T cell differentiation] (Ratio = 0.123, *p*-value < 0.0001), [GO:0050863_regulation of T cell activation] (Ratio = 0.099, *p*-value < 0.0001), [GO:0042110_T cell activation] (Ratio = 0.09, *p*-value < 0.0001); for CC: [GO:0042101_T cell receptor complex] (Ratio = 0.4, *p*-value < 0.0001), [GO:0001772_immunological synapse] (Ratio = 0.179, *p*-value < 0.0001), [GO:0005871_kinesin complex] (Ratio = 0.163, *p*-value < 0.0001); and for MF: [GO:0055131_C3HC4-type RING finger domain binding] (Ratio = 0.6, *p*-value < 0.0001), [GO:0035173_histone kinase activity] (Ratio = 0.25, *p*-value = 0.0001), [GO:0017080_sodium channel regulator activity] (Ratio = 0.161, *p*-value = 0.0001). The results obtained demonstrate an altered transcriptomic profile in mice after acute O_3_ exposure, involving a broad immune response and an unusually high level of differential expression of rhythmic genes.

### 3.2. Notch Signaling Pathway and Circadian Rhythm Significantly Upregulated After O_3_ Exposure

**Figure 2:**
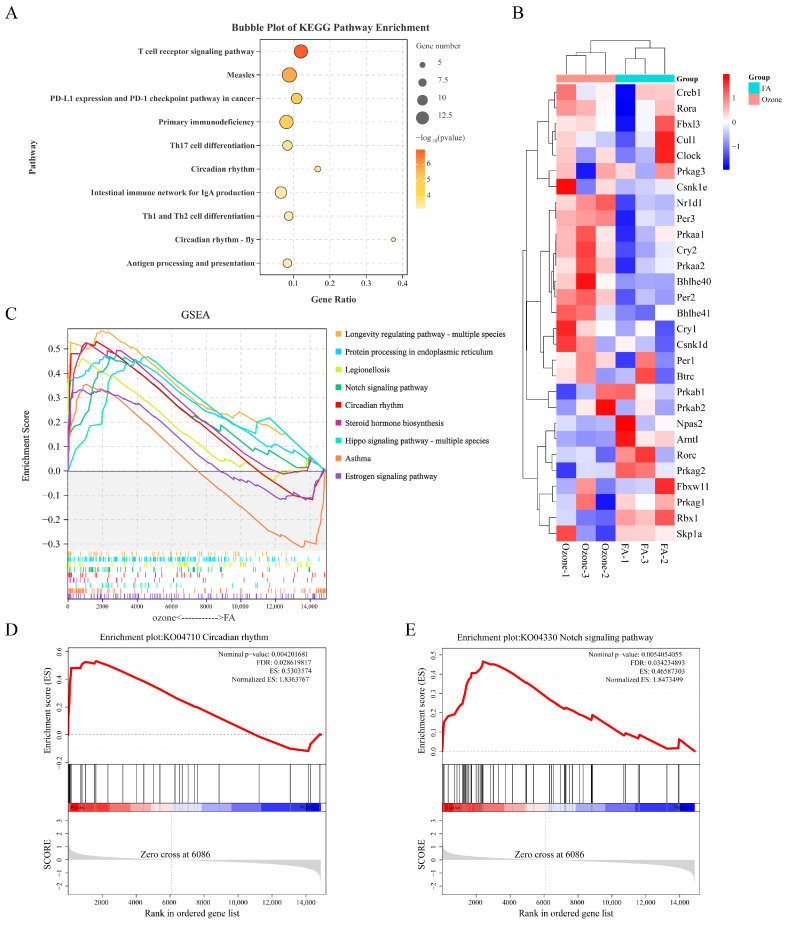
Enrichment Analysis of KEGG and GSEA. (**A**) KEGG pathway bubble plot. Axes: gene ratio (*x*), pathways (*y*). Bubble size: DEG count; color: −log_10_(*p*-value). (**B**) Enrichment profiles of 9 positively enriched pathways. Screening criteria: (|NES| > 1.5, FDR < 0.25, *p* < 0.05) (**C**) Heatmap of circadian rhythm pathway (ko04710) genes. Rows: genes; columns: samples (FA vs. Ozone); red/blue: high/low expression. (**D**) Detailed GSEA plot for circadian rhythm pathway (ko04710). Key metrics: NES = 1.84, FDR = 0.029; zero cross at rank 6086. (**E**) GSEA plot for NOTCH signaling pathway (ko04330). Metrics: NES = 1.85, FDR = 0.034; zero cross at rank 6086.

KEGG pathway analysis revealed concurrent activation of immune-related pathways and identified the circadian rhythm pathway (gene ratio = 0.167, *p* < 0.001) as a significantly altered non-immune process (Figure 2A), suggesting crosstalk between O_3_-triggered immune responses and circadian dysregulation. To mechanistically interpret prior functional enrichment results implicating circadian dysregulation, we performed GSEA on KEGG pathways. The analysis identified 9 significantly upregulated pathways (FDR < 0.25, |Normalized Enrichment Score (NES) | > 1.5, *p* < 0.05) (Figure 2B), including aberrant expression of the Circadian rhythm (ko04710) and Notch signaling pathways (ko04330). As demonstrated in Figure 2C, the circadian rhythm exhibited global transcriptional upregulation, with significant enrichment (NES = 1.84, FDR = 0.029), indicating activation. (Figure 2D). Similarly, Notch signaling showed robust enrichment (NES = 1.85, FDR = 0.034) (Figure 2E), suggesting synchronized pathway perturbation. These findings indicate a potential interplay between circadian rhythms and Notch signaling pathways in O_3_-induced lung transcriptome remodeling.

**Figure 3:**
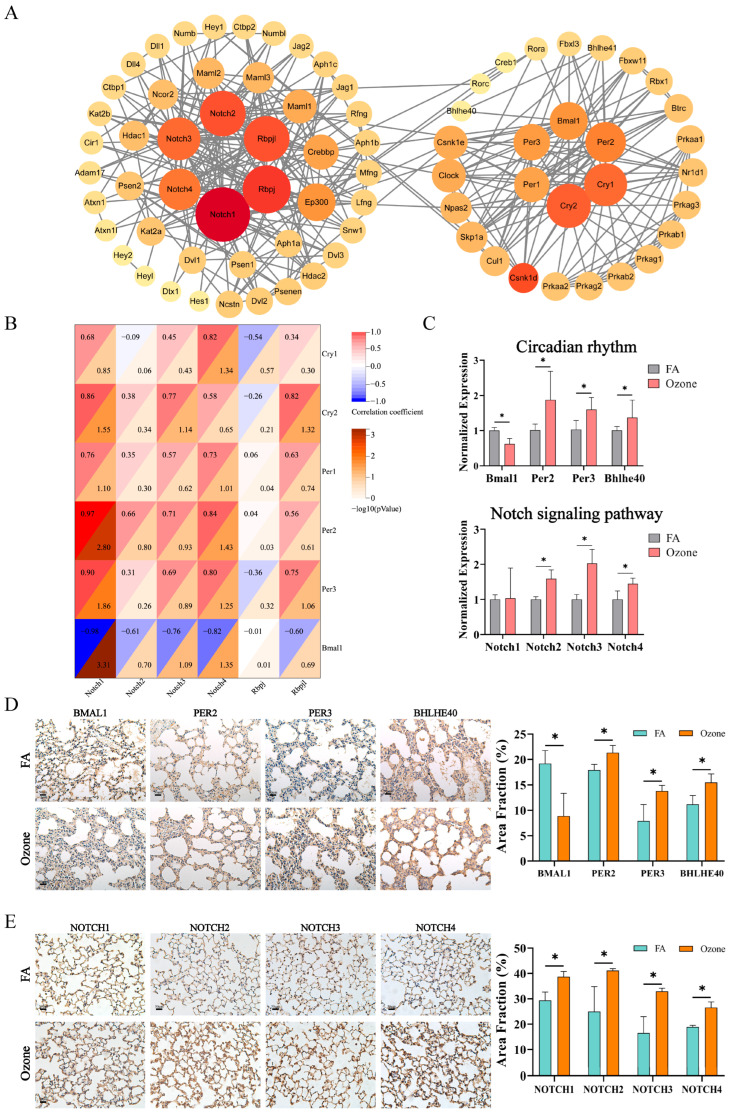
Integrated analysis of Notch-circadian rhythm crosstalk. (**A**) PPI network of enriched genes from Notch signaling (ko04330) and circadian rhythm (ko04710) pathways. Nodes sized/colored by degree centrality (STRING confidence > 0.9). (**B**) Correlation heatmap between core Notch (*Notch1/2/3/4*, *Rbpj*, *Rbpjl*) and circadian (*Cry1/2*, *Per1/2/3*, *Bmal1*) components. Upper triangle: Pearson r (red = positive, blue = negative); lower triangle: −log_10_(*p* -value). (**C**) RT-qPCR of *Bmal1*, *Bhlhe40*, *Per2*, *Per3* (upper) and *Notch1/2/3/4* (lower), normalized to FA controls (mean ± SD, * indicates *p* < 0.05 compared with FA, *n* = 6). (**D**) Representative IHC staining of BMAL1, PER2, PER3, BHLHE40 in FA/Ozone lung tissues (left; scale bars: 50 μm). Area fraction of positive staining (right) (mean ± SD, * indicates *p* < 0.05 compared with FA, *n* = 3). (**E**) Representative IHC staining of NOTCH1/2/3/4 in FA/Ozone lung tissues (left; scale bars: 50 μm). Area fraction of positive staining (right) (mean ± SD, * indicates *p*< 0.05 compared with FA, *n* = 3).

To further decipher the mechanistic crosstalk between Notch and circadian pathways implicated by prior analyzes, we conducted a PPI network analysis. Results showed that under the high-confidence filtering of the STRING database, there was a significant interaction between the circadian rhythm and Notch signaling pathway (Figure 3A). We further identified 12 high-degree nodes in the Notch signaling pathway (*Notch1/2/3/4*, *Rbpj*, *Rbpjl*; degree > 15) and circadian rhythm pathway (*Per1/2/3*, *Cry1/2*, *Bmal1*; degree > 12), and examined their gene expression correlations under ozone exposure conditions. Transcriptomic correlation analysis (Figure 3B) revealed distinct regulatory patterns: *Notch1* expression negatively correlated with *Bmal1* (r = −0.98, *p* < 0.05) but positively associated with *Per2* (r = 0.90, *p* < 0.05) and *Per3* (r = 0.97, *p* < 0.05). In contrast, *Notch4* showed negative correlation with *Bmal1* (r = −0.82, *p* < 0.05) but positive correlation with *Per3* (r = 0.84, *p* < 0.05) and *Cry1* (r = 0.82, *p* < 0.05). Additionally, *Rbpj* positively correlated with *Cry2* (r = 0.82, *p* < 0.05). The preceding analyzes demonstrated patterns of interference between pivotal proteins in the Notch signaling pathway and the circadian pathway subsequent to acute O_3_ exposure, thereby further substantiating the crosstalk between the two.

In consideration of the pivotal genes and their expression in the Notch and circadian signaling pathways, we proceeded to validate the genes *Bmal1*, *Per2*, *Per3*, *Bhlhe40*, and *Notch1/2/3/4* at the gene and protein levels. In a manner analogous to the sequencing results, exposure to O_3_ has been demonstrated to exert a significant effect on the expression of circadian rhythm genes in lung tissue. As demonstrated in Figure 3C, the RT-qPCR results indicated a decrease in the expression of *Bmal1* in the O_3_-exposed group, while the expression of *Bhlhe40*, *Per2*, and *Per3* increased in the O_3_-exposed group compared to the FA group (*p* < 0.05). Additionally, *Notch2*/*3*/*4* gene expression exhibited a significant increase following O_3_ exposure (*p* < 0.05). As demonstrated by Figure 3D, the results obtained from IHC staining revealed a significant decrease in BMAL1 expression within the alveolar region following O_3_ exposure, accompanied by a marked increase in the expression levels of PER2, PER3, and BHLHE40. These observations were corroborated through area fraction analysis of the IHC images, with a statistical significance of *p* < 0.05. Furthermore, a significant upregulation in the expression of signaling pathway proteins was observed in response to O_3_ exposure (Figure 3E; *p* < 0.05). The findings indicate that the outcomes of the validation process for genes and proteins are in alignment with the results of the correlation analyzes. Collectively, these results suggest that O_3_ exposure disrupts the normal expression of circadian rhythms and the Notch signaling pathway in lung tissue, and that there is a clear association between the two pathways.

### 3.3. O_3_ Exposure Leads to Altered Circadian Rhythm Expression Patterns in the Lungs After Notch3/4 Knockout

**Figure 4:**
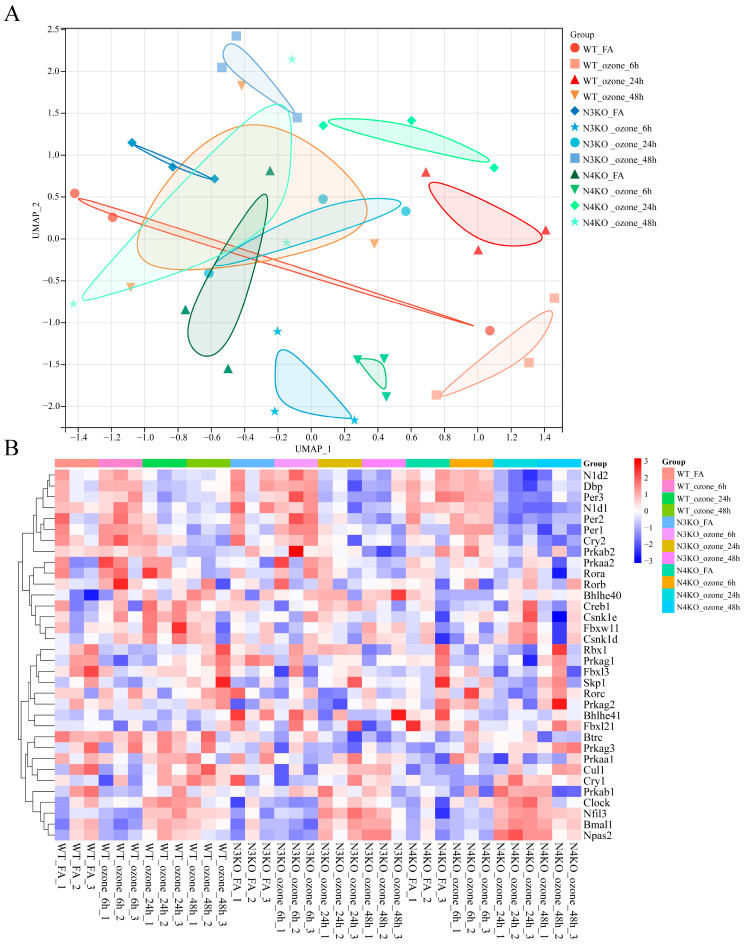
Transcriptomic profiling of O_3_-exposed wild-type and Notch-knockout mice. (**A**) UMAP of the unfiltered transcriptome from GSE58244 dataset. Groups include: Wild-type (WT) controls: FA, 6h/24h/48h ozone exposure; *Notch3*−/− (N3KO): FA., 6h/24h/48h ozone; *Notch4*−/− (N4KO): FA, 6h/24h/48h ozone (**B**) Heatmap of core circadian rhythm gene expression Rows: robust multiarray average (RMA) normalized expression; Row: Groups (the same as A), Column: Gene samples (from circadian rhythm pathway KO04710).

In order to explore the role of the Notch signaling pathway in the regulation of circadian rhythms after O_3_ exposure, the GEO database was queried for the dataset that best fit the purpose of the study (GSE58244). This contains gene expression matrices for the whole lung at different time points and genotypes after 0.3 ppm O_3_ exposure. To ensure clearer grouping of the dataset and distinguish it from previous studies, we classified and named the dataset as follows: mouse genotype_exposure factor_exposure time. Firstly, the transcriptomic landscape of O_3_-exposed mice with different genotypes was characterized using Uniform Manifold Approximation and Projection (UMAP) and heatmap analyzes of the dataset. The UMAP analysis (Figure 4A) involved the grouping of samples based on three factors: genotype, which included wild-type (WT), *Notch3*-knockout (N3KO), and *Notch4*-knockout (N4KO) categories; exposure condition, categorized as filtered air (FA) or O_3_; and post-exposure time course, which was subdivided into 6 h, 24 h, and 48 h time points. Distinct clustering patterns were observed, indicating that both genetic background and O_3_ exposure duration significantly influenced the transcriptomic profile. Specifically, WT samples, irrespective of exposure time, exhibited a clustering tendency that was distinct from the N3KO and N4KO samples. In the context of a uniform genetic background, exposure to O_3_ (at 6 h, 24 h, or 48 h) resulted in transcriptomic shifts when compared to the FA controls. These findings suggest the occurrence of O_3_-induced transcriptional responses. Furthermore, the N3KO and N4KO samples exhibited unique clustering patterns in response to O_3_ exposure, suggesting that *Notch3* and *Notch4* may have differential roles in regulating O_3_-responsive transcriptomics.

Heatmap-based visualization was employed to analyze the expression of core circadian rhythm genes (pathway KO04710) (Figure 4B). As demonstrated by the heatmap, there was a clear manifestation of disparate expression patterns across the various experimental groups. Hierarchical clustering of both genes and samples demonstrated that the expression of circadian rhythm genes was differentially regulated not only by genetic background but also by the duration of O_3_ exposure. Specifically, within the same genotype, O_3_ exposure for 6 h did not induce significant alterations in the expression of genes involved in the circadian rhythm. However, as the exposure duration extended, at 24 h and 48 h of O_3_ exposure, the expression patterns of circadian rhythm genes showed marked differences compared to both the FA and the 6 h O_3_ exposure groups. Furthermore, under identical O_3_ exposure conditions, the expression of circadian rhythm genes in N3KO and N4KO mice manifested a more intense color representation in the heatmap, indicating a more pronounced modulation of circadian rhythm gene expression in these Notch-knockout genotypes. Collectively, these findings suggest that *Notch3* and *Notch4* are involved in regulating the circadian rhythm pathway in the context of O_3_ exposure.

**Figure 5:**
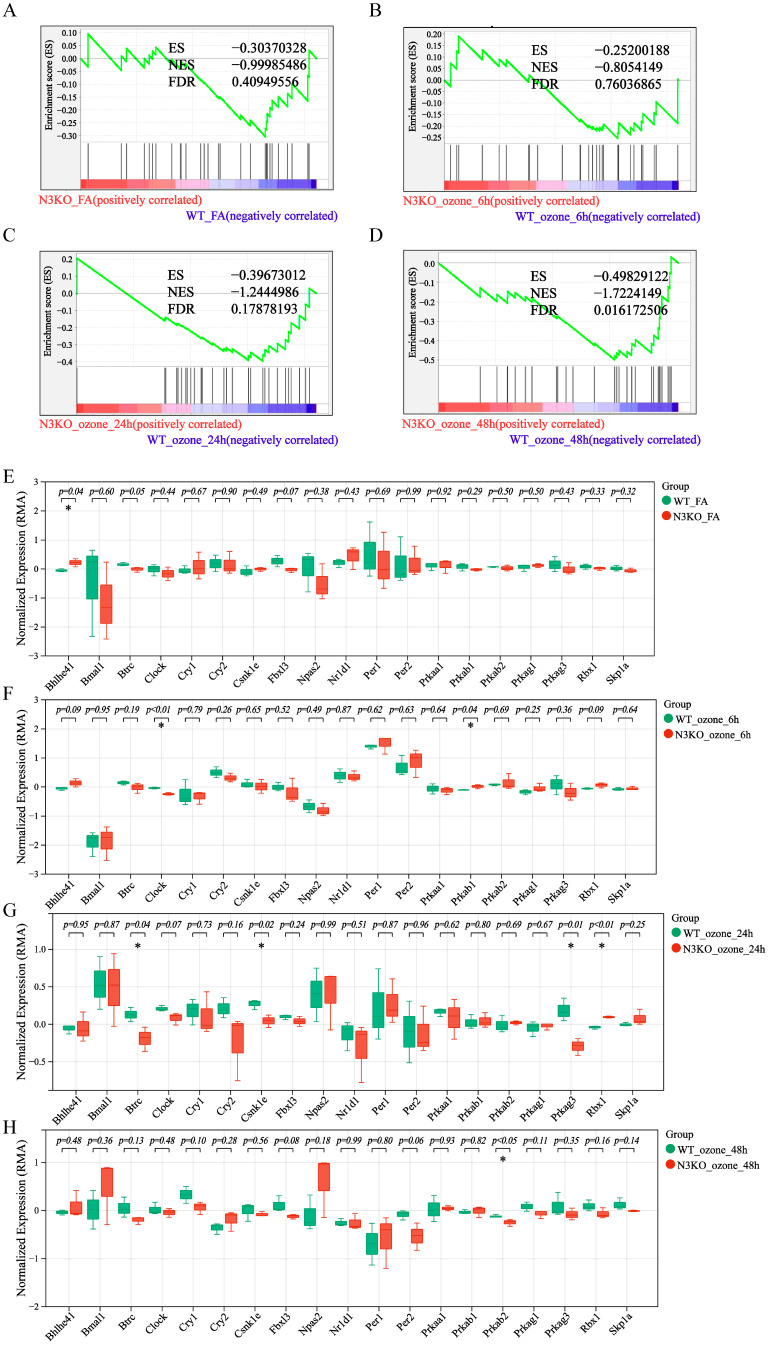
Alterations in the circadian rhythm pathway and core gene expression in *Notch3*-knockout mice exposed to O_3_. (**A**) GSEA of the KEGG circadian rhythm pathway (KO04710) in *Notch3*−/− mice versus wild-type controls under filtered air conditions (N3KO_FA vs. WT_FA, *n* = 6). (**B**) GSEA of the ko04710 pathway in *Notch3*−/− versus wild-type mice after 6 h O_3_ exposure (N3KO_ozone_6h vs. WT_ozone_6h, *n* = 6). (**C**) GSEA of the ko04710 pathway in *Notch3*−/− versus wild-type mice after 24 h O_3_ exposure (N3KO_ozone_24h vs. WT_ozone_24h, *n* = 6). (**D**) GSEA of the ko04710 pathway in *Notch3*−/− mice versus wild-type mice after 48 h O_3_ exposure (N3KO_ozone_48h vs. WT_ozone_48h, *n* = 6). (**E**) Box plots showing expression levels of circadian regulators N4KO_FA vs. WT_FA (* indicates *p* < 0.05 vs. WT_FA, *n* = 6). (**F**) Box plots of circadian gene expression at 6 h O_3_ exposure (* indicates *p* < 0.05 vs. WT_ozone_6h, *n* = 6). (**G**) Box plots of circadian gene expression at 24 h O_3_ exposure (* indicates *p* < 0.05 vs. WT_ozone_24h, *n* = 6). (**H**) Differential expression of circadian genes at 48 h O_3_ exposure (* indicates *p* < 0.05 vs. WT_ozone_48h, *n* = 6).

Under O_3_ exposure, *Notch3*-knockout (N3KO) mice exhibited distinct dysregulation of the circadian rhythm pathway compared to WT controls, as revealed by GSEA and gene expression profiling (Figure 5). In filtered air conditions (N3KO_FA vs. WT_FA) (Figure 5A), GSEA analysis of the KEGG circadian rhythm pathway (KO04710) showed no significant enrichment in N3KO mice (NES = −0.99985; FDR = 0.40950). Box plots of circadian regulators (Figure 5E) revealed that only *Bhlhe41* exhibited statistically significant differential expression between groups (*p* < 0.05), indicating non-significant systemic perturbation of circadian pathways in *Notch3*- knockout mice under unexposed conditions. Following 6 h O_3_ exposure (N3KO_ozone_6h vs. WT_ozone_6h) (Figure 5B), GSEA results demonstrated no global dysregulation of the circadian rhythm pathway (NES = −0.80541; FDR = 0.76037). However, box plots (Figure 5F) identified significant differences in the expression of core circadian regulators *Clock* and *Prkab1* (*p* < 0.05), suggesting localized disruption of circadian components despite the lack of pathway-level enrichment. After 24 h O_3_ exposure (N3KO_ozone_24h vs. WT_ozone_24h) (Figure 5C), GSEA revealed moderate but statistically trending dysregulation of the circadian pathway (NES = −1.24450; FDR = 0.17878). Box plots (Figure 5G) confirmed significant alterations in *Btrc*, *Csnk1e*, *Prkag3*, and *Rbx1* (*p* < 0.05), highlighting progressive O_3_-induced disruption of circadian regulatory networks in *Notch3*-knockout mice. With 48 h O_3_ exposure (N3KO_ozone_48h vs. WT_ozone_48h) (Figure 5D), GSEA indicated robust and persistent pathway perturbations (NES = −1.72241; FDR = 0.01617). Box plots (Figure 5H) further identified *Prkab2* as a significantly differentially expressed gene (*p* < 0.05). Collectively, these findings demonstrate that the circadian rhythm pathway in *Notch3* knockout mice exhibits dose-dependent sensitivity to O_3_ exposure, with prolonged exposure intensifying dysregulation and achieving statistical significance.

**Figure 6:**
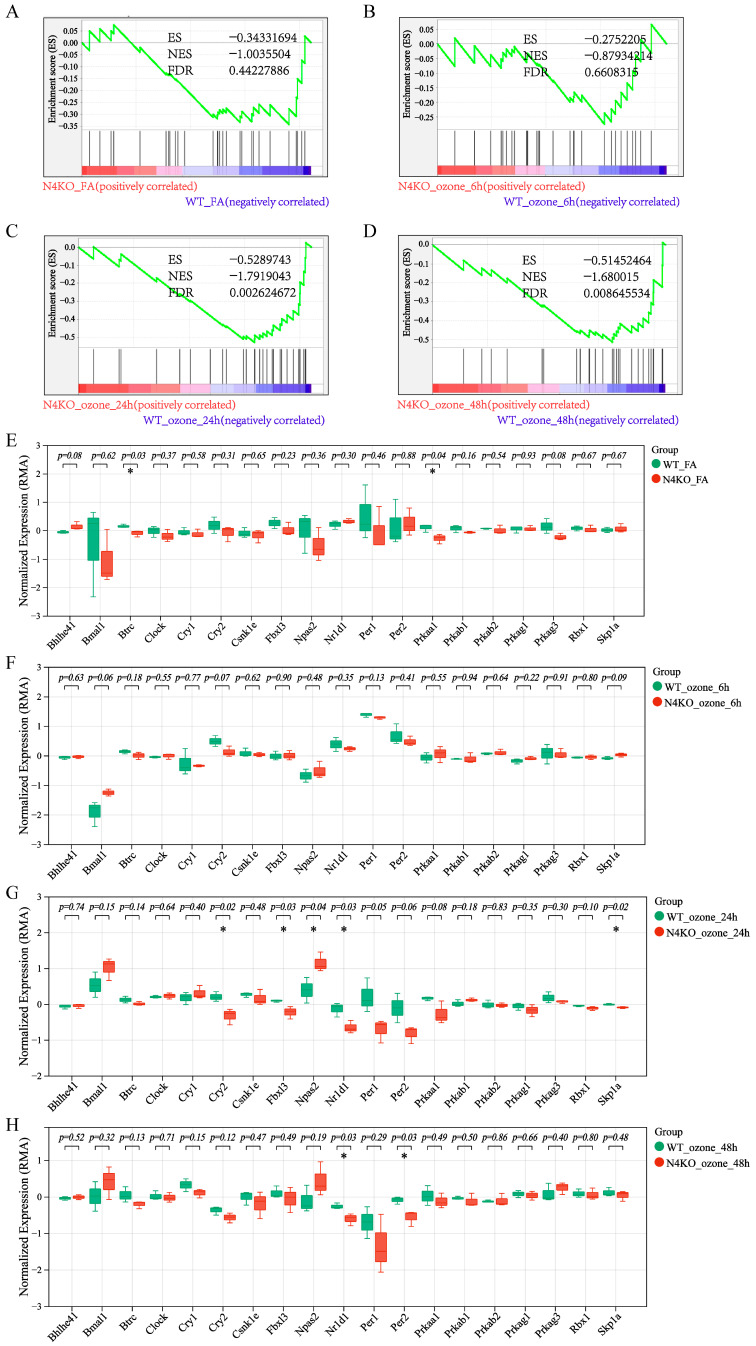
Dysregulation of the circadian rhythm pathway and core gene expression in *Notch4*-knockout mice under O_3_ exposure. (**A**) GSEA of circadian rhythm pathway (KO04710) in*Notch4*−/− (N4KO) vs. wild-type (WT) mice under filtered air (FA) (N4KO_FA vs. WT_FA, *n* = 6). (**B**) GSEA of KO04710 pathway in N4KO vs. WT mice after 6 h O_3_ exposure (N4KO_ozone_6h vs. WT_ozone_6h, *n* = 6). (**C**) GSEA of KO04710 pathway in N4KO vs. WT mice after 24 h O_3_ exposure (N4KO_ozone_24h vs. WT_ozone_24h, *n* = 6). (**D**) GSEA of KO04710 pathway in N4KO vs. WT mice after 48 h O_3_ exposure (N4KO_ozone_48h vs. WT_ozone_48h, *n* = 6). (**E**) Box plots of circadian regulator expression in N4KO_FA vs. WT_FA (* indicates *p* < 0.05, *n* = 6). (**F**) Box plots of circadian gene expression at 6 h O_3_ exposure (N4KO_ozone_6h vs. WT_ozone_6h; * indicates *p* < 0.05, *n* = 6). (**G**) Box plots of circadian gene expression at 24 h O_3_ exposure (N4KO_ozone_24h vs. WT_ozone_24h; * indicates *p* < 0.05, *n* = 6). (**H**) Box plots of circadian gene expression at 48 h O_3_ exposure (N4KO_ozone_48h vs. WT_ozone_48h; * indicates *p* < 0.05, *n* = 6).

*Notch4*-knockout (N4KO) mice also showed dysregulated circadian rhythm pathway activity under O_3_ exposure. In filtered air conditions (N4KO_FA vs. WT_FA) (Figure 6A), GSEA of the circadian rhythm pathway (KO04710) demonstrated pathway enrichment in N4KO mice (NES = −1.00355, FDR = 0.44227). Box plots revealed significant expression differences for *Btrc* and *Prkaa1* (*p* < 0.05) (Figure 6E), suggesting the presence of individual circadian rhythm gene alterations in *Notch4*- knockout mice, yet the overall pathway level changes are not significant. After 6 h O_3_ exposure (N4KO_ozone_6h vs. WT_ozone_6h) (Figure 6B), GSEA results supported pathway dysregulation. Although specific genes with significant changes were not as prominent as in other time points, the overall pathway enrichment indicated early O_3_-induced effects on circadian gene expression in N4KO mice. With 24 h O_3_ exposure (N4KO_ozone_24h vs. WT_ozone_24h) (Figure 6C), GSEA confirmed pathway enrichment. Box plots showed that *Cry2*, *Fbxl3*, *Npas2*, *Nr1d1*, and *Spk1a* had statistically significant expression changes (*p* < 0.05) (Figure 6G), reflecting complex circadian pathway disruptions in N4KO mice at this time point. Following 48 h O_3_ exposure (N4KO_ozone_48h vs. WT_ozone_48h) (Figure 6D), GSEA indicated continued pathway dysregulation. *Nr1d1* and *Per2* were significantly differentially expressed (*p* < 0.05) in box plots, emphasizing late-stage O_3_-induced alterations in circadian gene expression in *Notch4*-knockout mice (Figure 6H). Collectively, these results demonstrate that both *Notch3* and *Notch4* are involved in regulating the circadian rhythm pathway under O_3_ exposure, with distinct temporal and genotypic patterns of dysregulation.

## 4. Discussion

Although the association between O_3_ exposure and exacerbation of respiratory diseases has been extensively documented in animal models and epidemiological studies [8,41,42,43], and the phenomenon that air pollutants (including O_3_) can induce circadian rhythm disruption has also been preliminarily observed [23,44], the specific molecular pathways through which O_3_ perturbs the circadian rhythm of lung tissue remain elusive. The present study systematically reveals the critical regulatory role of the Notch signaling pathway (particularly *Notch3*/*4*) in O_3_-induced circadian rhythm disruption in lung tissue through a combination of approaches, including transcriptome analysis, multi-level validation, and re-analysis of public datasets.

Acute O_3_ exposure can synchronously induce inflammatory activation and systemic dysregulation of the circadian rhythm pathway in mouse lung tissue. Under normal light cycles, we simulated environmental exposure scenarios via acute O_3_ exposure (1.0 ppm, 3 h). Transcriptome analysis showed that the transcriptional profile of mouse lung tissue was characterized by the coexistence of significant inflammatory activation and circadian rhythm disruption. The universal activation of heat shock protein families (e.g., *Hspa1b*, *Hspa1a*) and immune-related pathways (e.g., T cell differentiation, immune synapse) confirmed the known pro-inflammatory effects of O_3_ [7,45]. Concurrently, core circadian rhythm genes exhibited characteristic dysregulation, namely the inhibition of *Bmal1* and upregulation of *Per2/3* and *Bhlhe40*, reflecting the imbalance of the transcription-translation feedback loop [46,47]. This pattern is consistent with previous observations that 1.0 ppm O_3_ exposure (3 h/day for 14 days) induces upregulation of rhythm genes *Per2/3* and downregulation of *Bmal1* [23]. Aberrant activation of *Bhlhe40* has also been confirmed in an O_3_-exposed lung parenchyma model [44], and it may inhibit the protein stability of the BMAL1-CLOCK complex through an autoregulatory loop, similar to its regulatory pattern in mouse hearts [48]. More importantly, GSEA analysis confirmed that this disruption is not a random fluctuation of individual genes but a systemic dysregulation of the entire circadian rhythm pathway (NES = 1.84, FDR = 0.029).

Circadian rhythm disruption is a potential mechanism underlying O_3_-induced pulmonary inflammation. Studies have shown that *Bmal1* or *Clock* deficiency may accelerate lung cell senescence and promote the progression of COPD [49]. Additionally, systemic or cell-specific *Bmal1* knockout impairs epithelial regeneration after influenza virus-induced lung injury [10], suggesting that the normal expression of core circadian rhythm genes is crucial for lung tissue repair and regeneration [11]. O_3_ exposure reduces the protein expression of NRF2, a protective factor against oxidative damage, while upregulating the pro-inflammatory marker IL-1β [50], thereby triggering an inflammatory cascade. Notably, BMAL1 can regulate *Nrf2* mRNA expression by directly binding to the E-box in the *Notch4* promoter, which in turn affects IL-1β production in macrophages [51]. This mechanism suggests that O_3_-induced circadian rhythm disruption (e.g., *Bmal1* downregulation) may act as a non-immune regulatory pathway for O_3_-induced inflammation by inhibiting NRF2-mediated antioxidant signaling and activating transcriptional regulation of IL-1β.

Beyond circadian rhythm disruption, aberrant activation of the Notch signaling pathway represents another core feature of O_3_-induced transcriptome remodeling in lung tissue, implying that it may serve as a key regulatory target in O_3_-induced lung injury. As a core pathway in lung injury repair [52], *Notch1/3* is highly expressed in COPD [53], pulmonary hypertension [54], and lung cancer [55], driving disease progression. In the present study, the Notch signaling pathway was significantly enriched after acute O_3_ exposure (NES = 1.85, FDR = 0.034), with synchronized upregulation of mRNA expression and alveolar-specific protein elevation (*p* < 0.05) of key receptors *Notch2/3/4*, confirming the systemic activation of Notch signaling in O_3_-exposed lung tissue. Previous studies have documented the universality of Notch signaling activation in response to air pollutant exposure: for example, O_3_ exposure activates *Notch3/4* expression, and their deficiency exacerbates O_3_-induced inflammation by inhibiting *Trim* and activating *Traf6* [36]. Furthermore, PM_2.5_ has been demonstrated to elicit the Notch signaling pathway through the accumulation of reactive oxygen species (ROS), thus contributing to the exacerbation of pulmonary fibrosis [56], suggesting that the Notch signaling pathway is a conserved responsive target in air pollutant-induced lung injury. Distinct from previous studies, our PPI network and correlation analysis revealed significant protein–protein interactions and expression associations between Notch family members and core circadian rhythm genes (*Bmal1*, *Per2/3*), indicating that the Notch signaling pathway also plays a critical role in O_3_-induced circadian rhythm disruption.

Re-analysis of GEO datasets further confirmed that *Notch3/4* are key regulators of O_3_-induced circadian rhythm disruption. Following 12–48 h of O_3_ exposure, a dose-dependent enhancement in the perturbation of the circadian rhythm pathway was observed in *Notch3/4* knockout mice. In contrast, only minor differences in individual circadian rhythm genes were detected in the filtered air (FA) control and 6 h O_3_ exposure groups. This highlights the dependence of *Notch3*/*4* regulatory effects on the duration of O_3_ exposure. In healthy lung tissue, *Notch3/4* deficiency had minimal impact on the overall circadian rhythm pathway but caused subtle changes in individual genes (e.g., *Notch3* deficiency reduced *Bhlhe41* expression, while *Notch4* deficiency upregulated *Btrc* and *Prkaa1*), which may be associated with basal regulation of pulmonary cell differentiation [57], phosphorylation [58], and ubiquitination pathways [59,60]. However, O_3_ exposure significantly amplified these perturbations: after 6 h of 0.3 ppm O_3_ exposure, *Notch3/4* deficiency altered the transcriptional profile of the circadian rhythm pathway, albeit without statistical significance (FDR > 0.05). In contrast, after 12–24 h of exposure, the perturbations became prominent. Specifically, in *Notch3*-knockout mice, reduced transcriptional activity of *Btrc* and *Csnk1e* (key regulators of *Per1* degradation) [61] may lead to PER1 protein accumulation, thereby interfering with PER/CRY complex-mediated negative feedback and ultimately disrupting CLOCK-BMAL1 transcriptional activity. Meanwhile, *Prkag2/3*, as γ subunits of AMPK [62], may weaken ubiquitination-mediated regulation of PER2 through ATP-dependent mechanisms, thereby affecting circadian rhythm gene expression [63]. Additionally, aberrant activation of *Rbx1* induced by *Notch3* deficiency participates in circadian rhythm regulation by catalyzing ubiquitination and degradation of PER and CRY [64]. In *Notch4*- knockout mice, downregulation of *Cry2/Per2* and upregulation of *Npas2* directly disrupted PER/CRY feedback inhibition and the activation balance of the NPAS2-BMAL1 complex [65]. Reduced *Nr1d1* expression further supported the overactivation of NPAS2-BMAL1 [66]. Furthermore, *Notch4* regulation of *Skp1a* and *Fbxl3* can directly modulate PER1/2 or CRY1/2 ubiquitination to regulate circadian rhythms [67,68]. Interestingly, although *Notch3*/*4* deficiency increased susceptibility to O_3_ exposure and circadian rhythm disruption, the perturbed circadian rhythm gene expression patterns still conformed to the transcription-translation feedback loop composed of BMAL1-CLOCK and PER/CRY. Therefore, the alterations in the single-time-point circadian rhythm gene profile induced by *Notch3*/*4* deficiency in this investigation are presumably responsible for the modification of the amplitude of circadian rhythm genes observed in normal lung tissue.

This study is the first to systematically reveal the regulatory role of *Notch3*/*4* in O_3_-induced circadian rhythm disruption in lung tissue. Our results demonstrate that O_3_ exposure can synchronously activate the Notch signaling pathway and disrupt the expression of core circadian rhythm genes, while *Notch3*/*4* play dose-dependent critical roles in maintaining circadian rhythm homeostasis under stress through regulating core circadian rhythm genes. The innovative value of this study lies in the first identification of the Notch-mediated regulatory mechanism underlying O_3_-induced circadian rhythm disruption, which expands the traditional understanding of air pollutant-induced injury mechanisms. Additionally, it provides a novel perspective for explaining the injury pattern involving pulmonary circadian rhythm disruption and inflammatory responses after O_3_ exposure. However, due to the complex hierarchical oscillatory network of the circadian rhythm system, single-time-point sampling cannot fully reflect the dynamic characteristics of rhythm perturbations. Moreover, direct binding evidence between Notch and circadian rhythm genes remains to be supplemented. Future studies can focus on “*Notch3*/*4*-targeted strategies to stabilize circadian rhythms” and further dissect the spatiotemporal characteristics of the “Notch-rhythm” crosstalk network through multi-time-point dynamic monitoring and molecular interaction validation, providing more refined theoretical basis for the precise prevention and treatment of air pollution-related lung diseases.

## 5. Conclusions

Notch signaling pathway, particularly *Notch3/4*, plays a key role in ozone-induced disruption of lung circadian rhythms, which establishes a novel mechanistic link between environmental pollutants and circadian rhythm gene alterations in respiratory diseases.

## Figures and Tables

**Table 1:** List of Primer Sequences.

Name	Sequences 5′-3′	
*Bmal1*	Forward	CTCAACCATCAGCGACTTCA
	Reverse	CCTTCCTTGGTGTTCTGCAT
*Per2*	Forward	AAAGCTGACGCACACAAAGAA
	Reverse	ACTCCTCATTAGCCTTCACCT
*Per3*	Forward	AACACGAAGACCGAAACAGAAT
	Reverse	CTCGGCTGGGAAATACTTTTTCA
*Bhlhe40*	Forward	CCGATTCTCCTCCATAGCCACT
	Reverse	ACCTCCAGGAAGCCATCAGACC
*Notch1*	Forward	CAGGCAATCCGAGGACTATG
	Reverse	CAGGCGTGTTGTTCTCACAG
*Notch2*	Forward	TGGTGGTCAGTGCATGGATAG
	Reverse	ATCTGGGGACACACATCGAC
*Notch3*	Forward	TGGCGACCTCACTTAAGACT
	Reverse	CACTGGCAGTTATAGGTGTTGAC
*Notch4*	Forward	CGAGGAAGATACGGAGTGGC
	Reverse	CTGCTCTGGTGGGCATACAT
*β-actin*	Forward	GGCCAACCGTGAAAAGATGA
	Reverse	CAGCCTGGATGGCTACGTACA

## Data Availability

The data presented in this study are available on request from the corresponding author.
